# Supplementary figures and images for: The spatial and temporal evolution of habitat quality and driving factors in nature reserves: a case study of 33 forest ecosystem reserves in Guizhou Province
Source: PeerJ. 2025 Mar 24;13:e19098. doi: 10.7717/peerj.19098 (PMC11949111; doi:10.7717/peerj.19098)

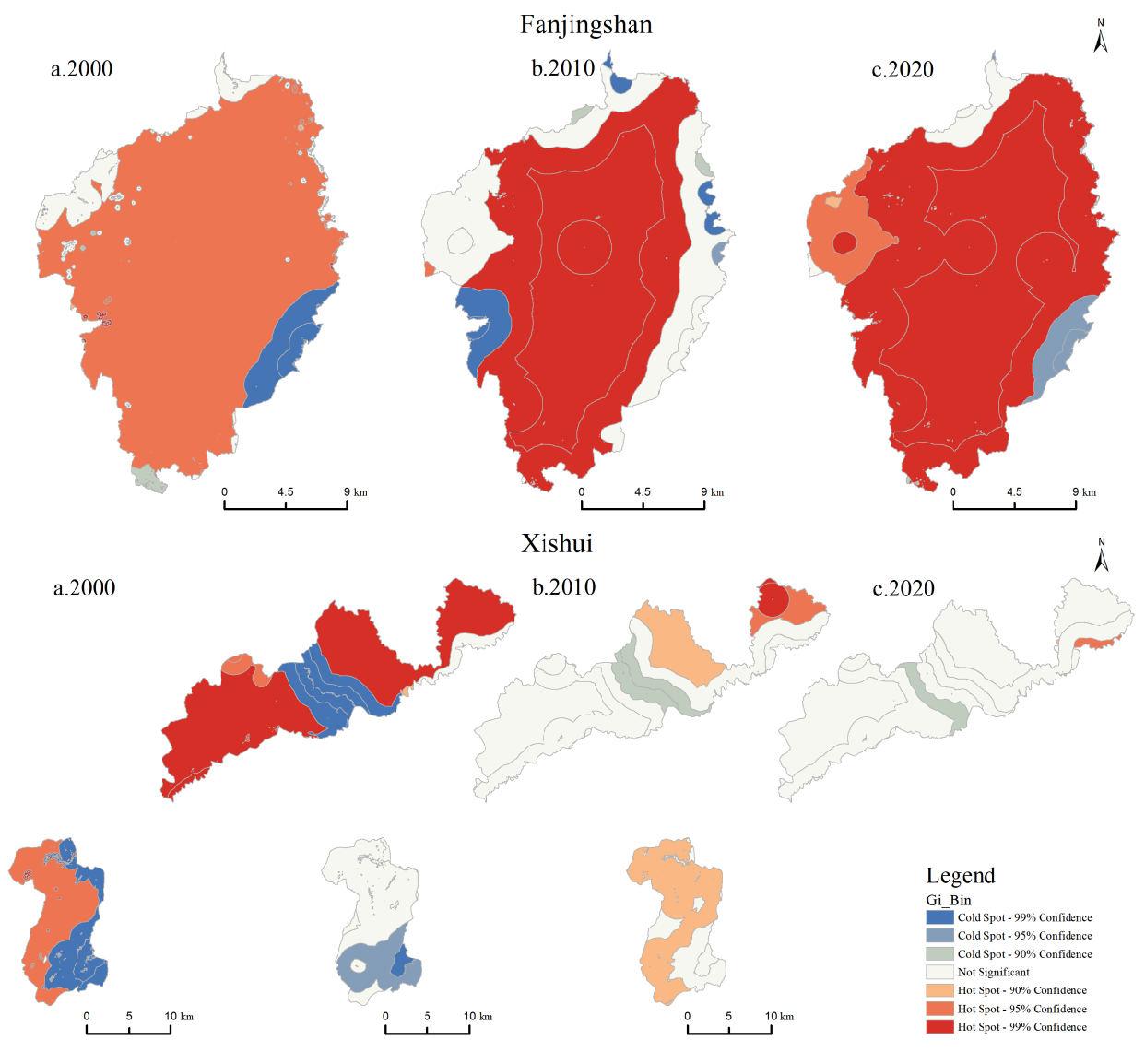

Supplement: Supplemental Information 2 [file peerj-13-19098-s002.png]

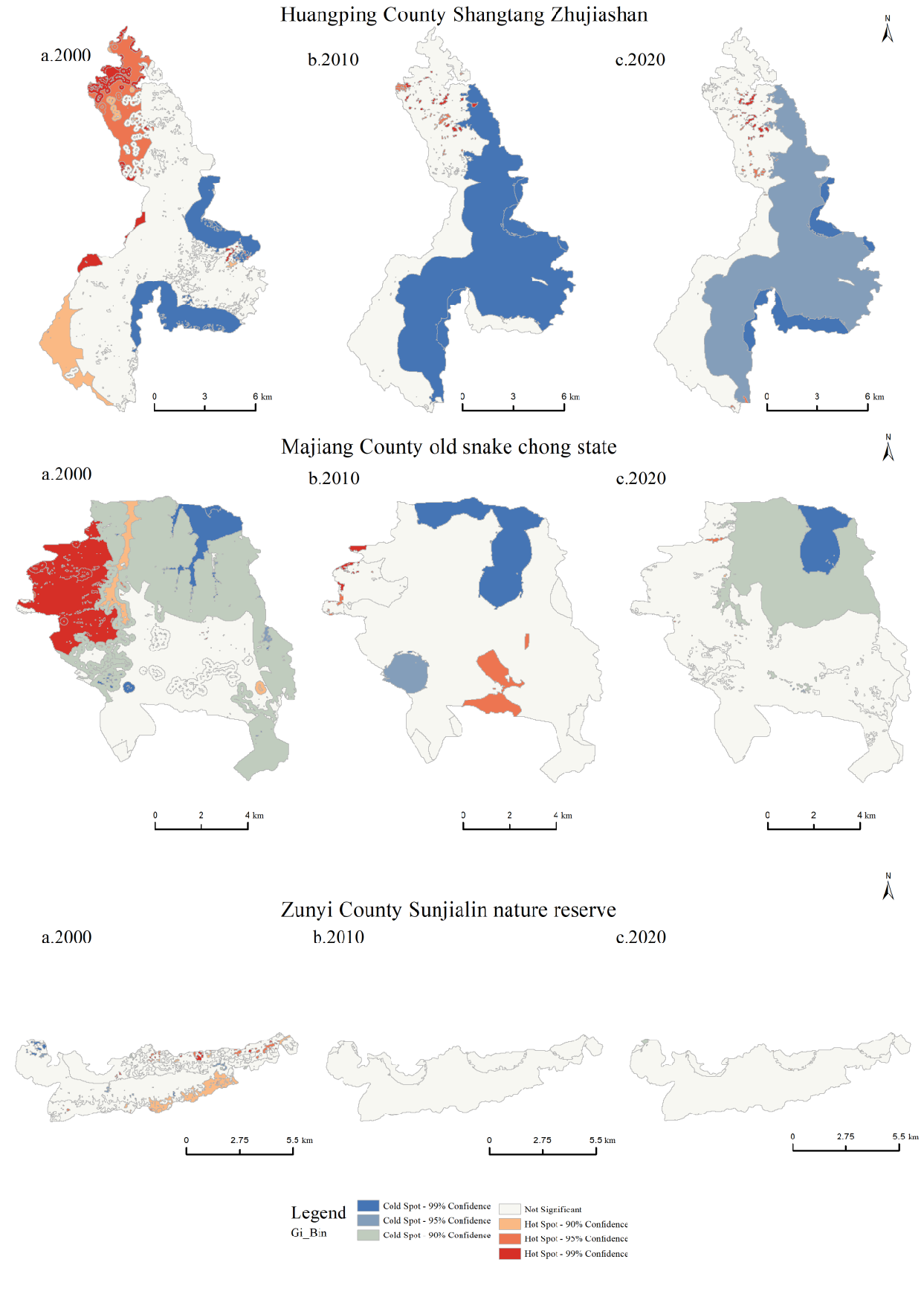

Supplement: Supplemental Information 3 [file peerj-13-19098-s003.zip › S2.png]

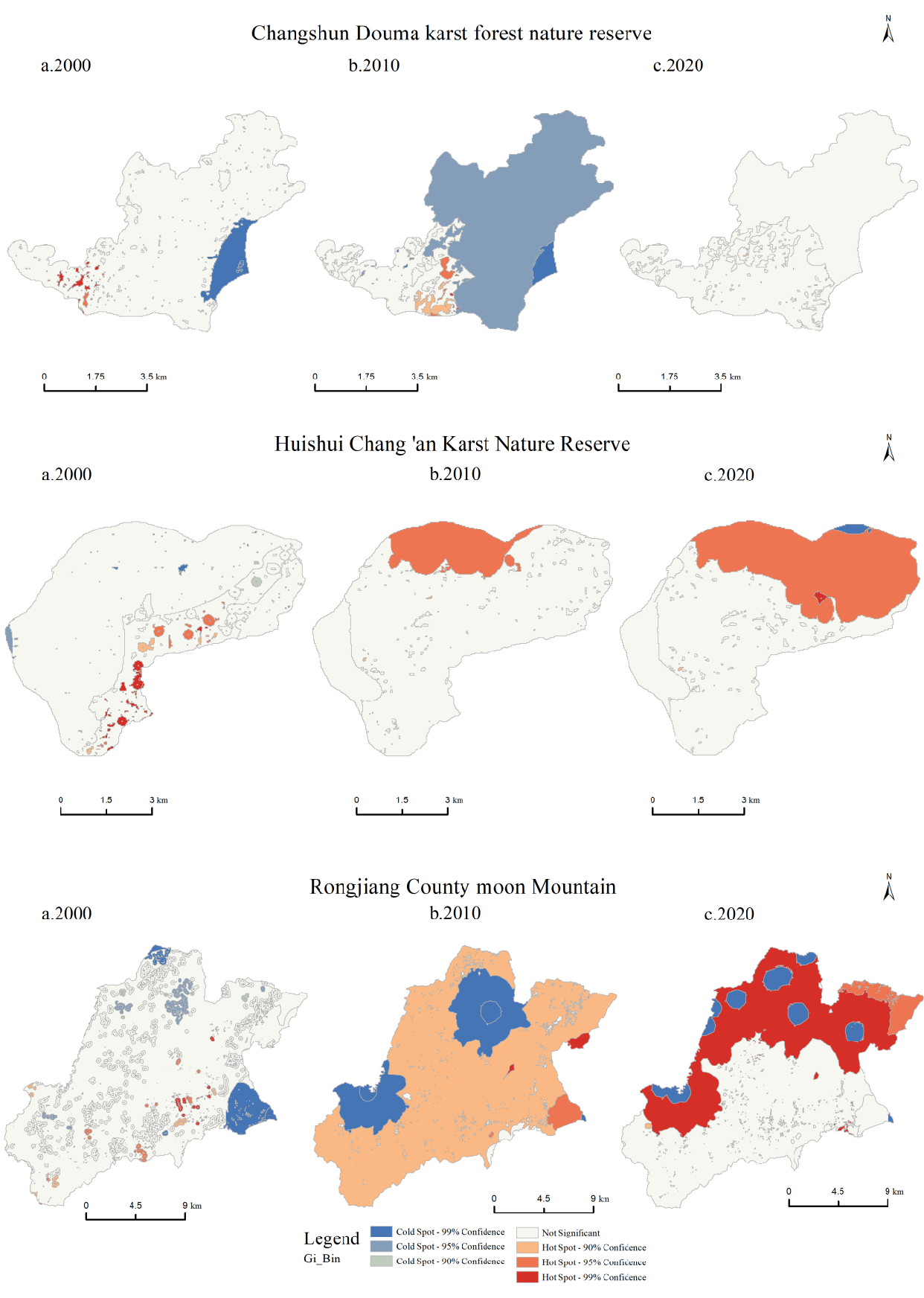

Supplement: Supplemental Information 3 [file peerj-13-19098-s003.zip › S3.png]
