# Supplementary material for: The spatial and temporal evolution of habitat quality and driving factors in nature reserves: a case study of 33 forest ecosystem reserves in Guizhou Province
Source: PeerJ. 2025 Mar 24;13:e19098. doi: 10.7717/peerj.19098 (PMC11949111; doi:10.7717/peerj.19098)
Supplement: Supplemental Information 5 [file peerj-13-19098-s005.docx]

| Supplement Table 1 Land use transfer matrix for nature reserves in Guizhou Province,2000-2020（ha） | | | | | | | | |
| --- | --- | --- | --- | --- | --- | --- | --- | --- |
| Time Period | Land Use Type | Cropland | Forest | Grassland | Impervious | Shrub | Water | Total |
| 2000-2010 | Cropland | 23642.91 | 4515.93 | 51.57 | 15.75 | 1333.17 | 6.57 | 29565.9 |
|  | Forest | 7395.3 | 487041.93 | 30.51 | 1.98 | 3076.11 | 0.09 | 497545.92 |
|  | Grassland | 105.39 | 76.59 | 115.29 | 0.45 | 70.29 | - | 368.01 |
|  | Impervious | - | - | - | 33.75 | - | 1.8 | 35.55 |
|  | Shrub | 615.69 | 2730.6 | 46.89 | - | 3910.05 | - | 7303.23 |
|  | Water | 6.48 | 3.78 | - | - | - | 48.51 | 58.77 |
|  | Total | 31765.77 | 494368.83 | 244.26 | 51.93 | 8389.62 | 56.97 | 534877.38 |
| 2010-2020 | Cropland | 24359.67 | 6629.13 | 45.18 | 73.35 | 588.6 | 69.84 | 31765.77 |
|  | Forest | 8614.89 | 482967.18 | 21.42 | 15.3 | 2745 | 5.04 | 494368.83 |
|  | Grassland | 121.41 | 32.67 | 65.25 | 1.08 | 23.31 | 0.54 | 244.26 |
|  | Impervious | - | - | - | 47.79 | - | 4.14 | 51.93 |
|  | Shrub | 1468.89 | 3346.11 | 31.05 | - | 3543.21 | 0.36 | 8389.62 |
|  | Water | 2.43 | 1.44 | - | 0.18 | - | 52.92 | 56.97 |
|  | Total | 34567.29 | 492976.53 | 162.9 | 137.7 | 6900.12 | 132.84 | 534877.38 |
| 2000-2020 | Cropland | 20531.34 | 8042.67 | 30.87 | 81.81 | 818.64 | 60.57 | 29565.9 |
|  | Forest | 12842.01 | 481110.57 | 60.39 | 22.68 | 3495.15 | 15.12 | 497545.92 |
|  | Grassland | 146.7 | 131.58 | 40.59 | 3.42 | 45.54 | 0.18 | 368.01 |
|  | Impervious | - | - | - | 29.7 | - | 5.85 | 35.55 |
|  | Shrub | 1044.18 | 3686.76 | 31.05 | - | 2540.79 | 0.45 | 7303.23 |
|  | Water | 3.06 | 4.95 | - | 0.09 | - | 50.67 | 58.77 |
|  | Total | 34567.29 | 492976.53 | 162.9 | 137.7 | 6900.12 | 132.84 | 534877.38 |
