# Supplementary material for: The spatial and temporal evolution of habitat quality and driving factors in nature reserves: a case study of 33 forest ecosystem reserves in Guizhou Province
Source: PeerJ. 2025 Mar 24;13:e19098. doi: 10.7717/peerj.19098 (PMC11949111; doi:10.7717/peerj.19098)
Supplement: Supplemental Information 6 [file peerj-13-19098-s006.docx]

|  | | | | | |  |  |  |  |  |  |  |
| --- | --- | --- | --- | --- | --- | --- | --- | --- | --- | --- | --- | --- |
| Year | 2000 | | | | 2010 | | | | 2020 | | | |
| Habitat Quality | HighestLevel | Higher Level | Lower Level | Lowest Level | HighestLevel | Higher Level | Lower Level | Lowest Level | HighestLevel | HigherLevel | Lower Level | Lowest Level |
|  | 0.75-1 | 0.5-0.75 | 0.25-0.5 | 0-0.25 | 0.75-1 | 0.5-0.75 | 0.25-0.5 | 0-0.25 | 0.75-1 | 0.5-0.75 | 0.25-0.5 | 0-0.25 |
| N1 | 82 | 16.1 | 1.87 | 0.03 | 10.32 | 78.09 | 10.5 | 1.09 | 0.48 | 67.12 | 29.49 | 2.92 |
| N2 | 69.85 | 22.99 | 6.98 | 0.18 | 36.88 | 44.24 | 14.73 | 4.14 | 31.03 | 45.25 | 19.08 | 4.64 |
| N3 | 87.08 | 11.45 | 1.44 | 0.03 | 37.87 | 48.33 | 12.36 | 1.44 | 33.6 | 37.56 | 24.73 | 4.11 |
| N4 | 88.56 | 10.76 | 0.68 | 0 | 64.84 | 30.86 | 4.05 | 0.24 | 57.1 | 35.45 | 7.04 | 0.42 |
| N5 | 45.52 | 45.63 | 8.82 | 0.03 | 3.46 | 72.52 | 22.74 | 1.28 | 3.71 | 47.6 | 44.21 | 4.48 |
| N6 | 35.12 | 50.22 | 14.04 | 0.61 | 0 | 65.72 | 29.78 | 4.5 | 0 | 59.39 | 35.66 | 4.95 |
| N7 | 60.8 | 35.76 | 3.44 | 0 | 5.07 | 61.79 | 29.87 | 3.27 | 2.45 | 54.45 | 35.87 | 7.24 |
| N8 | 0.84 | 32.35 | 57.52 | 9.29 | 1.86 | 45.46 | 47.26 | 5.42 | 0.84 | 32.35 | 57.52 | 9.29 |
| R1 | 84.95 | 14.08 | 0.97 | 0 | 3.29 | 76.64 | 18.96 | 1.11 | 0.25 | 59.09 | 38.47 | 2.19 |
| R2 | 73.04 | 24.78 | 2.18 | 0 | 0.1 | 76.32 | 22.09 | 1.49 | 0.07 | 53.47 | 40.43 | 6.04 |
| R3 | 24.35 | 66.6 | 8.94 | 0.12 | 0 | 57.01 | 41.35 | 1.65 | 0 | 24.78 | 70.31 | 4.91 |
| R4 | 7.05 | 73.99 | 18.96 | 0 | 0 | 61.54 | 38.45 | 0 | 0 | 50.43 | 49.56 | 0 |
| R5 | 11.62 | 72.92 | 15.4 | 0.06 | 0 | 26.48 | 70.17 | 3.36 | 0 | 10.39 | 78.96 | 10.64 |
| R6 | 67.94 | 29.05 | 3.01 | 0 | 1.68 | 83.26 | 14.99 | 0.07 | 0.78 | 81.1 | 17.74 | 0.38 |
| R7 | 28.06 | 62.97 | 8.82 | 0.16 | 0 | 51.86 | 44.67 | 3.48 | 0 | 26.54 | 66.62 | 6.84 |
| R8 | 0.02 | 42.93 | 53.41 | 3.65 | 0 | 6.67 | 73.46 | 19.87 | 0 | 0.12 | 78.55 | 21.33 |
| R9 | 21.52 | 62.48 | 15.94 | 0.07 | 0 | 48 | 45.31 | 6.69 | 0 | 34.3 | 56.78 | 8.92 |
| R10 | 14.39 | 66.71 | 18.42 | 0.48 | 0 | 39.36 | 53.86 | 6.78 | 0 | 38.86 | 54.26 | 6.87 |
| R11 | 42.22 | 48.75 | 8.86 | 0.16 | 0 | 61.23 | 33.73 | 5.04 | 0 | 9.63 | 74.49 | 15.88 |
| R12 | 5.62 | 72.43 | 21.84 | 0.1 | 0 | 45.4 | 52.48 | 2.12 | 0 | 25 | 72.81 | 2.19 |
| R13 | 14.94 | 66.77 | 17.59 | 0.71 | 0 | 23.98 | 67.31 | 8.72 | 0 | 6.94 | 69.63 | 23.43 |
| R14 | 38.31 | 46.28 | 15.41 | 0 | 0 | 74.11 | 25.89 | 0 | 0 | 36.3 | 58.7 | 5 |
| R15 | 0.78 | 89.89 | 9.22 | 0.11 | 0 | 18.99 | 78.83 | 2.17 | 0 | 38.28 | 61.73 | 0 |
| R16 | 20.19 | 78.93 | 0.89 | 0 | 0 | 76.44 | 23.55 | 0 | 0 | 52.08 | 47.22 | 0.7 |
| R17 | 21.51 | 73.77 | 4.72 | 0 | 0 | 55.33 | 44.32 | 0.35 | 0 | 50.59 | 48.98 | 0.42 |
| R18 | 0.13 | 84.55 | 15.32 | 0 | 0 | 23.92 | 75.03 | 1.05 | 0 | 20.33 | 77.12 | 2.55 |
| R19 | 7.05 | 73.99 | 18.96 | 0 | 0 | 61.54 | 38.45 | 0 | 0 | 50.43 | 49.56 | 0 |
| R20 | 43.26 | 46.64 | 10.1 | 0 | 0 | 89.23 | 10.76 | 0 | 0 | 86.34 | 13.66 | 0 |
| R21 | 0 | 58.53 | 41.45 | 0.01 | 0 | 0 | 87.66 | 12.34 | 0 | 0 | 82.09 | 17.91 |
| R22 | 63.39 | 36.44 | 0.17 | 0 | 0 | 87.44 | 12.55 | 0 | 0 | 49.36 | 49.46 | 1.18 |
| R23 | 1.89 | 84.41 | 13.66 | 0.04 | 0 | 33.97 | 61.17 | 4.85 | 0 | 0.02 | 77.63 | 22.36 |
| R24 | 21.73 | 71.76 | 6.51 | 0 | 0 | 48.36 | 50.9 | 0.74 | 0 | 28.67 | 70.24 | 1.09 |
| R25 | 2.46 | 89.36 | 8.18 | 0 | 0 | 26.9 | 72.41 | 0.7 | 0 | 11.37 | 87.91 | 0.73 |
|  |  |  |  |  |  |  |  |  |  |  |  |  |
|  |  |  |  |  |  |  |  |  |  |  |  |  |
