# Supplementary material for: The spatial and temporal evolution of habitat quality and driving factors in nature reserves: a case study of 33 forest ecosystem reserves in Guizhou Province
Source: PeerJ. 2025 Mar 24;13:e19098. doi: 10.7717/peerj.19098 (PMC11949111; doi:10.7717/peerj.19098)
Supplement: Supplemental Information 7 [file peerj-13-19098-s007.docx]

| Table 9 Global Moran Index of 33 Nature Reserves in Guizhou Province, 2000-2020 | | | | | | | |  |  |
| --- | --- | --- | --- | --- | --- | --- | --- | --- | --- |
| Year | 2000 | | | 2010 | | | 2020 | | |
| Code | Global Moran’I | Z-value | p-value | Global Moran’I | Z-value | p-value | Global Moran’I | Z-value | p-value |
| Average | 0.2816 | 50.2338 | 0 | -0.0998 | 11.8169 | 0 | 0.1107 | 11.1095 | 0 |
| N1 | 0.5293 | 24.3316 | 0 | -0.0378 | -1.1775 | 0.239 | -0.0869 | -2.5914 | 0 |
| N2 | 0.6774 | 14.4691 | 0 | 0.2072 | 2.9504 | 0 | 0.273 | 4.4643 | 0 |
| N3 | 0.325 | 7.1811 | 0 | 0.2366 | 5.0962 | 0 | 0.4969 | 7.1046 | 0 |
| N4 | 0.1761 | 1.2648 | 0.2059 | 0.7068 | 3.9381 | 0 | 0.5735 | 3.8913 | 0 |
| N5 | 0.2332 | 11.9998 | 0 | -0.3577 | -17.2418 | 0 | -0.0244 | -0.5985 | 0.5495 |
| N6 | 0.1338 | 6.4585 | 0 | -0.1906 | -7.89 | 0 | 0.0276 | 1.0927 | 0.2745 |
| N7 | 0.5334 | 14.0919 | 0 | 0.0953 | 1.9831 | 0.0474 | 0.319 | 5.9936 | 0 |
| N8 | 0.2455 | 3.4085 | 0 | 0.0697 | 1.1984 | 0.2307 | 0.2455 | 3.4085 | 0 |
| R1 | 0.4352 | 15.5609 | 0 | 0.0637 | 2.0889 | 0.0367 | 0.0192 | 0.6409 | 0.5216 |
| R2 | 0.294 | 13.3075 | 0 | -0.1255 | -6.9931 | 0 | 0.2045 | 7.8318 | 0 |
| R3 | 0.2369 | 11.7819 | 0 | -0.3915 | -16.2945 | 0 | -0.0469 | -1.4271 | 0.1535 |
| R4 | -0.0997 | -2.5058 | 0.0122 | -0.3594 | -9.5019 | 0 | -0.4865 | -9.3475 | 0 |
| R5 | -0.0582 | -3.6127 | 0 | -0.1003 | -2.5262 | 0.0115 | 0.2033 | 3.4107 | 0 |
| R6 | 0.377 | 14.984 | 0 | -0.0534 | -1.5263 | 0.1269 | -0.0728 | -2.2366 | 0.0253 |
| R7 | 0.2201 | 7.8926 | 0 | -0.2915 | -8.834 | 0 | -0.0498 | -1.1245 | 0.2608 |
| R8 | 0.0379 | 2.1818 | 0.0291 | 0.2923 | 6.9127 | 0 | -0.0873 | -1.6344 | 0.1015 |
| R9 | 0.4156 | 8.1035 | 0 | 0.0546 | 1.0698 | 0.2847 | 0.036 | 0.8829 | 0.3773 |
| R10 | 0.2993 | 10.8373 | 0 | 0.2611 | 5.6386 | 0 | 0.2022 | 4.6635 | 0 |
| R11 | 0.5165 | 14.832 | 0 | -0.3119 | -7.5208 | 0 | 0.244 | 2.9295 | 0 |
| R12 | 0.0257 | 1.2194 | 0.2227 | -0.3218 | -10.9779 | 0 | -0.2042 | -4.7823 | 0 |
| R13 | 0.0293 | 0.7874 | 0.431 | 0.1312 | 1.8508 | 0.0642 | 0.2129 | 1.8954 | 0.058 |
| R14 | 0.0974 | 3.5546 | 0 | -0.4342 | -14.5235 | 0 | 0.103 | 2.294 | 0.0218 |
| R15 | 0.2194 | 7.1503 | 0 | -0.2525 | -3.8412 | 0 | -0.4061 | -8.3276 | 0 |
| R16 | 0.4114 | 7.6785 | 0 | -0.3782 | -8.8051 | 0 | -0.1854 | -3.4933 | 0 |
| R17 | 0.3692 | 5.1227 | 0 | 0.1082 | 1.8589 | 0.063 | -0.1296 | -1.8694 | 0.0616 |
| R18 | -0.3433 | -8.0882 | 0 | 0.0322 | 0.485 | 0.6276 | 0.0325 | 0.1105 | 0.6596 |
| R19 | -0.0997 | -2.5058 | 0.0122 | -0.3594 | -9.5019 | 0 | -0.4965 | -9.3475 | 0 |
| R20 | 0.1069 | 2.266 | 0.0234 | -0.5084 | -13.701 | 0 | -0.4358 | -10.0682 | 0 |
| R21 | -0.4646 | -10.4336 | 0 | -0.5352 | -3.2968 | 0 | -0.6825 | -4.0341 | 0 |
| R22 | 0.2383 | 2.2913 | 0.0219 | 0 | 0.3743 | 0.7082 | 0.2473 | 2.1103 | 0.0348 |
| R23 | -0.0453 | -0.2188 | 0.8268 | 0.2764 | 1.8188 | 0.0689 | -0.1497 | -1.0424 | 0.2972 |
| R24 | -0.1905 | -2.1405 | 0.0323 | -0.2577 | -3.1558 | 0 | -0.1905 | -2.1405 | 0.0323 |
