# Supplementary material for: The spatial and temporal evolution of habitat quality and driving factors in nature reserves: a case study of 33 forest ecosystem reserves in Guizhou Province
Source: PeerJ. 2025 Mar 24;13:e19098. doi: 10.7717/peerj.19098 (PMC11949111; doi:10.7717/peerj.19098)
Supplement: Supplemental Information 8 [file peerj-13-19098-s008.docx]

Table S4 Interaction results of Habitat Quality factors in national Nature Reserves

|  | N1 | N2 | N3 | N4 | N5 | N6 | N7 | N8 |
| --- | --- | --- | --- | --- | --- | --- | --- | --- |
| 2000 | X4∩X7(0.5854) | X1∩X5(1.0000) | X1∩X7(0.7772) | X6∩X7(0.7898) | X1∩X5(0.7696) | X1∩X5(0.8116) | X1∩X5(1.0000) | X3∩X5(0.6575) |
|  | X3∩X7(0.5802) | X2∩X5(1.0000) | X3∩X7(0.7708) | X3∩X7(0.6957) | X3∩X5(0.7632) | X3∩X5(0.8097) | X2∩X5(1.0000) | X4∩X5(0.6449) |
|  | X1∩X6(0.5604) | X3∩X5(1.0000) | X4∩X7(0.7627) | X1∩X7(0.6778) | X5∩X7(0.7566) | X5∩X7(0.7909) | X3∩X5(1.0000) | X1∩X5(0.6431) |
|  | X4∩X6(0.5552) | X4∩X5(1.0000) | X6∩X7(0.7544) | X4∩X7(0.6427) | X5∩X6(0.7177) | X4∩X5(0.7902) | X4∩X5(1.0000) | X4∩X7(0.6294) |
| 2010 | X4∩X6(0.5959) | X1∩X5(1.0000) | X4∩X7(0.7128) | X1∩X2(0.5510) | X4∩X5(0.5709) | X3∩X7(0.6788) | X1∩X5(1.0000) | X4∩X6(0.6982) |
|  | X3∩X6(0.5479) | X2∩X5(1.0000) | X3∩X7(0.7087) | X3∩X2(0.5464) | X3∩X5(0.5661) | X3∩X5(0.6671) | X2∩X5(1.0000) | X4∩X7(0.6977) |
|  | X1∩X6(0.5676) | X3∩X5(1.0000) | X4∩X6(0.6918) | X6∩X2(0.5452) | X3∩X6(0.5599) | X1∩X5(0.6591) | X3∩X5(1.0000) | X3∩X6(0.6908) |
|  | X4∩X7(0.5675) | X4∩X5(1.0000) | X1∩X7(0.6754) | X6∩X7(0.5288) | X1∩X5(0.5510) | X4∩X7(0.6535) | X4∩X5(1.0000) | X1∩X7(0.6848) |
| 2020 | X6∩X7(0.2529) | X1∩X5(1.0000) | X1∩X7(0.7502) | X1∩X7(0.7166) | X3∩X6(0.5201) | X3∩X7(0.6957) | X1∩X5(1.0000) | X4∩X6(0.7041) |
|  | X2∩X7(0.2488) | X2∩X5(1.0000) | X3∩X7(0.7442) | X3∩X7(0.6887) | X4∩X6(0.5080) | X1∩X7(0.6770) | X2∩X5(1.0000) | X3∩X6(0.7037) |
|  | X5∩X7(0.1954) | X3∩X5(1.0000) | X3∩X6(0.7114) | X2∩X7(0.6592) | X1∩X6(0.4896) | X4∩X7(0.6759) | X3∩X5(1.0000) | X4∩X6(0.6910) |
|  | X2∩X5(0.0943) | X4∩X5(1.0000) | X4∩X7(0.7041) | X4∩X7(0.6373) | X4∩X7(0.4725) | X6∩X7(0.6668) | X4∩X5(1.0000) | X3∩X7(0.6910) |
