# Supplementary material for: The spatial and temporal evolution of habitat quality and driving factors in nature reserves: a case study of 33 forest ecosystem reserves in Guizhou Province
Source: PeerJ. 2025 Mar 24;13:e19098. doi: 10.7717/peerj.19098 (PMC11949111; doi:10.7717/peerj.19098)
Supplement: Supplemental Information 9 [file peerj-13-19098-s009.docx]

Table S5 Interaction results of Habitat Quality factors in local-level Nature Reserves

| Year | R1 | R2 | R3 | R4 | R5 | R6 | R7 | R8 |  |
| --- | --- | --- | --- | --- | --- | --- | --- | --- | --- |
| 2000 | X1∩X5(1.0000) | X5∩X7(0.8742) | X5∩X7(0.6660) | X3∩X7(0.6171) | X1∩X5(0.7616) | X5∩X7(0.7701) | X3∩X4(0.4700) | X1∩X5(0.8391) |  |
|  | X2∩X5(1.0000) | X5∩X1(0.8543) | X5∩X6(0.6576) | X4∩X7(0.5906) | X5∩X7(0.7544) | X1∩X7(0.3505) | X3∩X6(0.4274) | X3∩X5(0.8263) |  |
|  | X3∩X5(1.0000) | X5∩X4(0.8479) | X5∩X3(0.6476) | X1∩X7(0.5811) | X3∩X5(0.7482) | X3∩X7(0.3324) | X1∩X4(0.4269) | X6∩X5(0.8071) |  |
|  | X4∩X5(1.0000) | X5∩X3(0.8228) | X5∩X1(0.6421) | X4∩X6(0.5275) | X5∩X6(0.7409) | X4∩X7(0.3057) | X3∩X7(0.4198) | X7∩X5(0.8064) |  |
| 2010 | X1∩X5(1.0000) | X6∩X4(0.4078) | X5∩X6(0.5242) | X3∩X4(0.6196) | X5∩X7(0.5151) | X6∩X7(0.5090) | X3∩X7(0.4238) | X4∩X5(0.6117) |  |
|  | X2∩X5(1.0000) | X6∩X3(0.3968) | X5∩X4(0.5006) | X3∩X7(0.5698) | X5∩X6(0.4609) | X6∩X5(0.4099) | X3∩X4(0.3996) | X3∩X5(0.5751） |  |
|  | X3∩X5(1.0000) | X1∩X7(0.3926) | X5∩X3(0.4868) | X1∩X7(0.5653) | X5∩X3(0.4510) | X4∩X7(0.3993) | X3∩X6(0.3791) | X7∩X5(0.5725) |  |
|  | X4∩X5(1.0000) | X1∩X6(0.3922) | X5∩X7(0.4831) | X3∩X6(0.4959) | X5∩X4(0.4534) | X1∩X7(0.3902) | X4∩X7(0.3480) | X1∩X5(0.5547) |  |
| 2020 | X1∩X5(1.0000) | X1∩X7(0.5131) | X5∩X3(0.3494) | X3∩X7(0.5483) | X5∩X7(0.5386) | X6∩X7(0.4902) | X3∩X4(0.4636) | X3∩X5(0.4431） |  |
|  | X2∩X5(1.0000) | X6∩X4(0.4726) | X5∩X4(0.3049) | X1∩X7(0.5256) | X2∩X7(0.5339) | X6∩X5(0.3925) | X3∩X2(0.4382) | X4∩X5(0.4387) |  |
|  | X3∩X5(1.0000) | X3∩X7(0.4440) | X5∩X6(0.2994) | X4∩X7(0.4941) | X4∩X7(0.5296) | X1∩X7(0.3808) | X3∩X7(0.4251) | X7∩X5(0.4065) |  |
|  | X4∩X5(1.0000) | X1∩X6(0.4429) | X2∩X4(0.2911) | X1∩X4(0.4801) | X3∩X7(0.5192) | X4∩X6(0.3540) | X3∩X6(0.4215) | X1∩X5(0.3982) |  |
| Year | R9 | R10 | R11 | R12 | R13 | R14 | R15 | R16 |  |
| 2000 | X4∩X6(0.7113) | X5∩X4(0.7081) | X5∩X7(0.7606) | X5∩X7(0.8688) | X5∩X7(0.7372) | X5∩X7(0.8889) | X5∩X7(0.7068) | X4∩X6(0.5411) |  |
|  | X4∩X2(0.6858) | X3∩X7(0.6954) | X5∩X4(0.7452) | X5∩X3(0.8341) | X5∩X3(0.7101) | X5∩X6(0.8889) | X5∩X4(0.6677) | X4∩X7(0.5088) |  |
|  | X1∩X6(0.6719) | X5∩X7(0.6899) | X5∩X6(0.7375) | X5∩X4(0.8089) | X5∩X1(0.6997) | X5∩X3(0.8621) | X5∩X3(0.6462) | X4∩X5(0.4111) |  |
|  | X3∩X6(0.6719) | X5∩X3(0.6828) | X5∩X1(0.7329) | X5∩X1(0.8005) | X5∩X6(0.6749) | X5∩X4(0.8426) | X5∩X6(0.6421) | X6∩X7(0.3939) |  |
| 2010 | X4∩X6(0.7371) | X3∩X7(0.7315) | X7∩X6(0.6946) | X5∩X6(0.6101) | X4∩X7(0.7229) | X5∩X7(0.8889) | X3∩X7(0.4937) | X4∩X7(0.4252) |  |
|  | X3∩X6(0.7174) | X6∩X7(0.6944) | X7∩X4(0.6762) | X5∩X4(0.5796) | X1∩X7(0.7193) | X5∩X6(0.8889) | X4∩X7(0.4289) | X3∩X7(0.4219) |  |
|  | X4∩X7(0.6893) | X5∩X7(0.6938) | X7∩X1(0.6682) | X5∩X7(0.5600) | X1∩X7(0.7141) | X5∩X3(0.8621) | X6∩X7(0.4217) | X4∩X5(0.3938) |  |
|  | X3∩X6(0.6828) | X1∩X7(0.6887) | X7∩X5(0.6525) | X5∩X1(0.5570) | X3∩X7(0.7092) | X5∩X4(0.8426) | X3∩X5(0.4036) | X4∩X6(0.3839) |  |
| 2020 | X3∩X6(0.6879) | X3∩X7(0.7287) | X3∩X4(0.6731) | X5∩X7(0.5578) | X1∩X7(0.8308) | X6∩X7(0.6185) | X5∩X7(0.6438) | X2∩X7(0.9192) |  |
|  | X4∩X6(0.6723) | X1∩X7(0.7127) | X6∩X7(0.6158) | X1∩X7(0.5632) | X3∩X7(0.8190) | X4∩X7(0.6062) | X3∩X7(0.5533) | X2∩X6(0.9118) |  |
|  | X4∩X7(0.6478) | X5∩X7(0.6744) | X3∩X7(0.6030) | X5∩X6(0.5330) | X4∩X7(0.8168) | X5∩X7(0.6005) | X4∩X7(0.5494) | X2∩X4(0.9092) |  |
|  | X3∩X6(0.6405) | X4∩X7(0.6741) | X3∩X6(0.5650) | X7∩X6(0.5163) | X6∩X7(0.8083) | X1∩X7(0.5999) | X1∩X7(0.5376) | X4∩X7(0.9072) |  |
| Year | R17 | R18 | R19 | R20 | R21 | R22 | R23 | R24 | R25 |
| 2000 | X5∩X7(0.7416) | X5∩X7(0.8128) | X5∩X3(0.9605) | X5∩X6(0.9811) | X5∩X4(0.8962) | X6∩X7(0.8918) | X4∩X6(0.8401) | X4∩X5(0.8402) | X3∩X5(0.7653) |
|  | X4∩X7(0.6585) | X5∩X4(0.7440) | X5∩X4(0.9556) | X5∩X7(0.9810) | X5∩X7(0.8944) | X4∩X7(0.8729) | X3∩X6(0.8236) | X3∩X5(0.6515) | X4∩X5(0.7466) |
|  | X3∩X7(0.6501) | X5∩X6(0.7196) | X5∩X6(0.9305) | X5∩X4(0.9760) | X5∩X1(0.8799) | X3∩X7(0.8688) | X6∩X7(0.8058) | X5∩X6(0.6321) | X5∩X7(0.7447) |
|  | X3∩X1(0.5749) | X5∩X3(0.6801) | X5∩X1(0.9247) | X5∩X3(0.9753) | X5∩X4(0.8673) | X1∩X7(0.7951) | X3∩X7(0.7681) | X5∩X7(0.6017) | X5∩X6(0.6096) |
| 2010 | X3∩X7(0.7479) | X5∩X7(0.6911) | X5∩X3(0.8212) | X5∩X7(0.9323) | X5∩X6(0.6231) | X4∩X7(0.8608) | X6∩X7(0.8662) | X4∩X5(0.6646) | X4∩X5(0.8050) |
|  | X4∩X7(0.7419) | X4∩X7(0.6323) | X5∩X4(0.8072) | X5∩X4(0.9096) | X4∩X6(0.5961) | X3∩X7(0.8174) | X3∩X6(0.8488) | X3∩X5(0.6202) | X4∩X7(0.7576) |
|  | X1∩X7(0.7180) | X6∩X7(0.6259) | X5∩X6(0.7604) | X5∩X3(0.8845) | X3∩X6(0.5956) | X4∩X6(0.8126) | X4∩X7(0.8455) | X6∩X7(0.5967) | X3∩X5(0.7570) |
|  | X1∩X4(0.6916) | X3∩X7(0.5934) | X5∩X1(0.6822) | X5∩X7(0.8439) | X4∩X5(0.5898) | X1∩X7(0.7960) | X4∩X6(0.8420) | X4∩X6(0.5510) | X3∩X6(0.7537) |
| 2020 | X5∩X7(0.7669) | X4∩X6(0.6581) | X5∩X4(0.8182) | X5∩X7(0.9092) | X7∩X3(0.6532) | X3∩X7(0.8221) | X3∩X7(0.8257) | X3∩X6(0.7272) | X4∩X5(0.8980) |
|  | X1∩X7(0.6492) | X6∩X7(0.6399) | X5∩X6(0.8089) | X5∩X4(0.8645) | X1∩X3(0.6372) | X4∩X7(0.8221) | X7∩X6(0.8091) | X3∩X4(0.6729) | X3∩X5(0.8906) |
|  | X3∩X5(0.6358) | X3∩X6(0.5970) | X5∩X3(0.7356) | X5∩X3(0.8479) | X6∩X3(0.6367) | X2∩X3(0.7402) | X4∩X7(0.7629) | X3∩X1(0.6559) | X5∩X7(0.8136) |
|  | X3∩X7(0.6350) | X5∩X6(0.5946) | X5∩X1(0.6221) | X5∩X6(0.8421) | X2∩X3(0.5937) | X2∩X4(0.7402) | X1∩X7(0.7582) | X4∩X6(0.5664) | X6∩X7(0.7810) |
|  |  |  |  |  |  |  |  |  |  |
